# Social media, vaccine hesitancy and trust deficit in immunization programs: a qualitative enquiry in Malappuram District of Kerala, India

**DOI:** 10.1186/s12961-021-00698-x

**Published:** 2021-08-11

**Authors:** Anoop T. Nair, Kesavan Rajasekharan Nayar, Shaffi Fazaludeen Koya, Minu Abraham, Jinbert Lordson, Chitra Grace, Sreekutty Sreekumar, Priya Chembon, Kamala Swarnam, A. Marthanda Pillai, Anant Kumar Pandey

**Affiliations:** 1 Primary Health Center Chaliyar, Department of Health, Malappuram, Kerala India; 2 Global Institute of Public Health, Trivandrum, Kerala India; 3 Boston University School of Public Health, Boston, MA USA; 4 Ministry of Health, Al Taif, Makkah, Saudi Arabia; 5 Ananthapuri Hospitals and Research Institute, Trivandrum, Kerala India; 6 Xavier Institute of Social Service (XISS), Ranchi, Jharkhand India

**Keywords:** Vaccine resistance, Vaccine hesitancy, Trust deficit, Anti-vaccine messages, Social media

## Abstract

**Background:**

With increased penetration of the internet and social media, there are concerns regarding its negative role in influencing parents’ decisions regarding vaccination for their children. It is perceived that a mix of religious reasons and propaganda by anti-vaccination groups on social media are lowering the vaccination coverage in Malappuram district of Kerala. We undertook a qualitative study to understand the factors responsible for generating and perpetuating vaccine hesitancy, the pathways of trust deficit in immunization programs and the interaction between various social media actors.

**Methods:**

In-depth interviews and focus group discussions were conducted among parents/caregivers, physicians, public sector health staff, alternative system medical practitioners, field healthcare workers and teachers in areas with highest and lowest vaccination coverage in the district, as well as with communication experts.

**Results:**

The trust deficit between parents/caregivers and healthcare providers is created by multiple factors, such as providers’ lack of technical knowledge, existing patriarchal societal norms and critical views of vaccine by naturopaths and homeopaths. Anti-vaccine groups use social media to influence caregivers' perceptions and beliefs. Religion does not appear to play a major role in creating vaccine resistance in this setting.

**Conclusions:**

A long-term, multipronged strategy should be adopted to address the trust deficit. In the short to medium term, the health sector can focus on appropriate and targeted vaccine-related communication strategies, including the use of infographics, soft skills training for healthcare workers, technical competency improvement through a mobile application-based repository of information and creation of a media cell to monitor vaccine-related conversations in social media and to intervene if needed.

**Supplementary Information:**

The online version contains supplementary material available at 10.1186/s12961-021-00698-x.

## Background

An emerging body of evidence suggests that anti-vaccination campaigns within social media and by some alternative system medical practitioners, including naturopaths and homeopaths, negatively influence immunization [1–3]. Deficiency of trust in both healthcare providers and mainstream medicine is a major determinant of resistance to vaccination [4]. Vaccine hesitancy refers to the delay in acceptance or refusal of vaccines despite the availability of vaccination services [5]. In contrast, the term ‘vaccine resistance’ refer to a ‘conscious decision to refuse the recommended vaccination’, or ‘the arguments critical of vaccination policy’, and implies a collective action, at times stemming from a ‘fundamental opposition to the dominant biomedical understanding of health and disease’ [6, 7]

The level of knowledge possessed by health workers on vaccines and their confidence in their ability to communicate effectively to parents about vaccines are important factors which can influence vaccine acceptance, along with trust, attitudes and beliefs [8]. However, the emergence of social media enables people with anti-vaccine beliefs to generate, consume and share information [9].

The South Indian state of Kerala has significantly reduced mortality and morbidity due to vaccine-preventable diseases (VPDs) and has brought down infant mortality to 6 per 1000 live births [10]. However, VPDs are re-emerging in Malappuram District in northern Kerala. In 2016, 229 cases of diphtheria were reported from the district, including two deaths [11, 12]. As per the National Family Health Survey (NFHS) 2015–2016, there were 82% fully immunized children in Kerala, with the corresponding figure for Malappuram being 70.6%; there was also a well pronounced urban and rural difference (61.1% and 80. 2%, respectively) [13]. The decline in full immunization coverage in the latter district was accompanied by a decrease in the coverage of most individual vaccines. Coverage in urban areas was consistently lower than in rural areas. Another recent survey found that of the 342 657 children aged < 5 years in Malappuram district, 23 912 were not fully immunized and that 36% of children aged 5–10 years had received no immunizations at all [13].

Kerala is the first digital state in India, and it has the highest mobile penetration (> 30 million connections for a population of 33 million), with an internet penetration rate of 37% [14, 15]. The municipality of Malappuram offers free Wi-Fi to its citizens [16]. However, there is increasing concern among public health workers and administrators that access to social media is increasingly influencing vaccination decisions, similar to what has been reported in other contexts experiencing a similar upsurge in social media use [17, 18]. In Malappuram, this is particularly worrying in the context of lower vaccination coverage [19, 20].

One major constraint in dealing with anti-vaccine social media messages is the inadequate capacity among health workers to respond to these messages, as reported in recent literature [21, 22]. However, this has not been studied in the context of Kerala, which has a well-functioning health system comparable to that found in some developed countries. The factors responsible for the resistance and trust deficit are not clearly understood in the context of social media usage. A clearer understanding of the interaction between various social media actors and the pathways involved in generating this trust deficit is needed in order to technically empower the health workers.

Therefore, the aim of this study was to explore the factors responsible for generating and perpetuating vaccine hesitancy and mistrust in the immunization programme in Malappuram district in the context of emerging anti-vaccine social media campaigns and to understand how the trust deficit between caregivers and health workers influences caregivers’ decisions on child vaccination.

## Methods

To collect information we used qualitative methods, specifically in-depth interviews (IDIs) and focus group discussions (FGDs). The objective was to gain an understanding of the social and health system factors in the context of increased social media usage and how social media messages contribute to the development of vaccine hesitancy and mistrust in the immunization programme. In addition, we also performed a quantitative content analysis of the vaccine-related Information Education and Communication (IEC) materials and anti-vaccine YouTube videos; the results of this latter analysis will be published separately.

The study was conducted in four health subcentre (HSC) areas of Nilambur taluk in Malappuram district of Kerala state (India). These HSCs were selected from the catchment areas of two primary health centres (PHC), one of which had the highest vaccination coverage (98.9%) in the area and the other had the lowest (79%). Each of thsee PHCs had five HSCs, and we randomly selected two HSCs that fell within the administration of each PHC.

Malappuram is the most populous district in the state (4.11 million; 2011 census [23]). The Muslim majority (70.24%) district has a literacy rate of 93.57% (males: 95.76%, females: 91.62%) [23] and a total of 124 public health institutions, including 20 community health centers (CHCs), 20 PHCs that are always open and 66 other PHCs [24].

### Study participants

The IDIs were conducted among doctors of various disciplines (modern medicine, homeopathy and naturopathy), schoolteachers, religious leaders (Muslim, Hindu and Christian) and communication campaign experts. Separate FGDs were conducted with caregivers (mother, father, grandfather or grandmother) of vaccinated children and with partially vaccinated/unvaccinated children, modern medicine doctors, public health nurses and Accredited Social Health Activist (ASHA) workers. We defined children (aged 0–2 years) as partially vaccinated when they missed one or more National Immunization Programme (NIP) vaccines for the age, as unvaccinated when they missed all vaccinations for their age in the NIP and as vaccinated when they had received all vaccinations as per the NIP.

### Data collection

Prior to data collection, permission was obtained from the Government of Kerala and the District Medical Officer (DMO), Malappuram. For IDIs with providers, religious heads and schoolteachers, respondents were selected through purposive sampling with the help of Panchayath (local government) and health authorities in the respective study areas. Face-to-face IDIs were conducted at their offices, ensuring privacy and confidentiality. Communication campaign experts were identified through the websites of national and international health organizations, and IDIs were conducted through emails after receiving consent. FGDs with healthcare workers were conducted in the respective PHC or HSC. Using the list of vaccination status of children aged < 2 years obtained from the PHCs as a sample frame, we identified houses with vaccinated, partially vaccinated or unvaccinated children and invited consecutive caregivers for interviews. In case of refusals, the next eligible household in the list was included and this process was continued until 8–12 respondents in each FGD group agreed to participate. FGDs with caregivers of vaccinated and partially vaccinated/unvaccinated children were conducted separately at Anganwadi (village level pre-school childcare) centres. All IDIs and FGDs were audio recorded without any personal identifiers. Data were collected between November 2017 and March 2018 up to the point at which it was felt that saturation was achieved as no new themes or trends were emerging.

### Data analysis

After transcription and translation, KRN and SS analysed the content. The responses were repeatedly read to identify frequently reported patterns with similarity and differences. These transcripts along with field notes were used to code and categorize in order to conceptualize the conversations into themes. These two investigators also read the information and field notes in order to cross-check and finalize the themes and they prepared an abridged transcript related to the research question. A process of triangulation was attempted by constantly referring to the field notes prepared by the researchers. KRN and SS performed this entire process manually, and the emergent themes were discussed with other authors to refine the themes. This process enabled the authors to identify the three main themes, namely social factors, service delivery factors and factors related to health workers, as discussed in detail in the following sections.

## Results

In total, we held 34 IDIs and 22 FGDs with 252 respondents. IDIs were held with government and private physicians (*n* = 4), homeopathic and naturopathic practitioners (*n* = 8), schoolteachers (*n* = 4), religious heads (*n* = 12), district health officials (*n* = 2) and communication experts (*n * = 4). The FGDs were conducted among caregivers of vaccinated (*n* = 8) and unvaccinated children (*n* = 8), private physicians (*n* = 2), public health nurses (*n* = 2) and ASHA workers (*n* = 2). Details of the respondents are given in Table 1.

**Table 1:** Details of study participants

Qualitative method of information collection	Category of participants	Participants
In-depth interviews (*n* = 34 participants, among whom* n *= 14 women)	Government physicians	Two; one per study area, both women
	Private physicians	Two; one per study area, both men
	Homeopathic practitioners	Four; two per study area, three women
	Naturopathic practitioners	Four; two per study area, all women
	School teachers	Four; two per study area, two men and two women
	Religious heads	All men Muslim- Four, two per study area Hindu- Four, two per study area Christian- Four, two per study area
	Communication experts	Three men of a total of four
	District officers	Two females; one medical and one immunization officer
Focus group discussion (*n* = 218 participants, among whom* n* = 128 women) (1 FGD per group per study area)	Caregivers of vaccinated children	Mothers: 12 participants per FGD Fathers: 12 in FGD 1 and 8 in FGD 2 Grandfathers: 10 in FGD 1 and 9 in FGD 2 Grandmothers: 10 participants per FGD
	Caregivers of unvaccinated children	Mothers: 12 in FGD 1 and 8 in FGD 2 Fathers: 10 participants per FGD Grandfathers: 8 in FGD 1 and 12 in FGD 2 Grandmothers: 11 in FGD 1 and 9 in FGD 2
	Private physicians	8 in FGD 1 and 9 in FGD 2
	Public health nurses	8 in FGD1 and 9 in FGD 2
	ASHA workers	11 in FGD 1 and 10 in FGD 2

*FGD* Focus group discussion

The IDIs and FGDs were analyzed thematically to determine the factors leading to the trust deficit between caregivers and health workers with respect to vaccination in the context of the emergence of social media campaigns. Three main themes emerged from the analysis: (1) personal factors (religion, patriarchal societal structure of the study area); (2) the presence of an anti-vaccine group (including certain individuals practicing alternative systems of medicine; influence of the anti-vaccine views of international anti vaccine groups); (3) health system factors (lack of trust in the health system by caregivers and the inability of the health system staff to address the doubts and concerns of the caregivers regarding vaccination).

The complete set of themes and narratives are available as Additional file 1 in this article. The three themes were categorized into sub-themes and are summarized below with a few selected relevant quotes.

### Social factors

#### Issues related to faith and religion

Most caregivers were of the opinion that religion does not play a negative role in the decision to vaccinate. However, many providers suggested that certain communities or sects within the Muslim and Christian religions do not believe in taking medications or being vaccinated.

“They (religious leaders) usually tell us to take vaccination.”

(Mother of unvaccinated child, FGD, study area 2).

“A group of Muslims do not believe in medicines and treatment. For them, Allah is the one who gives diseases to the child and if the child dies, it’s Allah’s destiny. They ask why they should try to change that destiny by using vaccines.”

(FGD, ASHAs, study area 1).

#### Patriarchy

Mothers of unvaccinated children opined that husbands usually make the decision to vaccinate the children or not, even if the mother is willing to allow the child to be vaccinated. An interesting feature that emerged is that the male heads of many of these families work in Middle East countries where they seem to be have been negatively influenced by anti-vaccine social media messages.

“The main decision makers of the family are fathers… The only media they have access to…is social media like WhatsApp…. They are mostly non-resident Keralites…. Newspapers and television are the main source of positive messages on vaccination. Social media is mostly spreading negative messages on vaccination.”

(Fathers of vaccinated children, FGD, study area 1).

#### Past negative experiences and doubts

Negative experiences with regard to vaccination were found to be a major concern of caregivers. They also have doubts regarding the need for vaccination for a healthy child.

“My child was very active before vaccination. After that particular vaccination, he couldn’t move his leg. This is the main reason for not taking my younger child for vaccination.”

(Father of unvaccinated child, FGD, study area 1).

“We need to treat only when the child is affected with disease. What is the point in taking prior medication?”

(Mother of unvaccinated child, FGD, study area 2).

#### Access to internet and social media

Many respondents said that they are exposed to both positive and negative vaccine-related messages through social media like WhatsApp, Facebook and YouTube videos which make them confused regarding which decision to make. The anti-vaccine groups propagate negative messages focused on “horrifying side effects" of vaccination, depopulation, international lobbying and the presence of harmful contents in vaccines.

“Which view are we, the less educated people, supposed to believe? If he (anti vaccine naturopath) is spreading false information, the government should take proper action. Then we will have more trust in the government health department.”

(Mother of unvaccinated child, FGD, study area 2)

“Negative messages get more visibility and circulation at all times.”

(Communication Expert, IDI).

### Service delivery factors

#### Lack of trust by caregivers

Many respondents refused vaccination because of their lack of trust in the allopathic system of medicine. Also, they were concerned about vaccine administration procedures.

“Regarding vaccination, I have no trust in allopathy. I follow homeopathy which is giving good results.”

(Father of unvaccinated child, study area 1)

“We don’t know whether they are keeping vaccines in ice boxes or not. I doubt the quality”.

(Father of unvaccinated child, FGD, study area 2).

#### Factors related to health workers

Field-level health workers themselves expressed their lack of information and training to respond to the queries and doubts posed by caregivers, especially in response to negative messages in social media. A lack of coordination between different systems of medicine with regard to vaccination is another issue.

“We find it difficult to convince the laymen about the science behind vaccination. It is a very complex one and we are not trained.”

(ASHA, FGD, Study area 1).

## Discussion

**Fig. 1:**
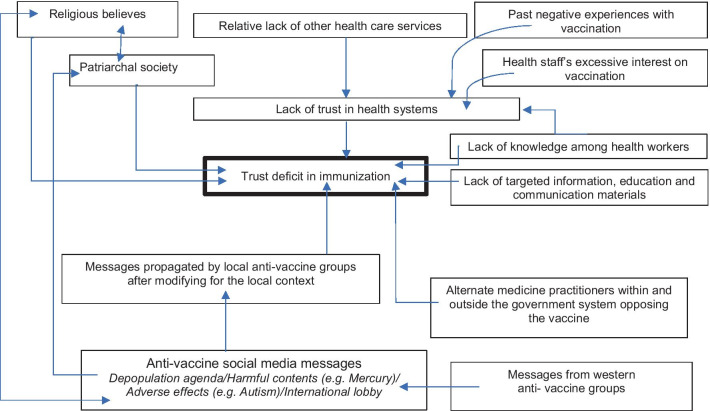
Pathways in the process of generating trust deficit in immunization programme

Based on the findings, we developed a framework to explain the trust deficit of caregivers in the district of Malappuram (Fig. 1). As compared to the Complacency, Convenience and Confidence (3Cs) model and the 2014 World Health Organization–Strategic Advisory Group of Experts on Immunization (SAGE) group Determinants of Vaccine Hesitancy Matrix report [25], our data suggest social media usage as a key factor in the framework which drives vaccine hesitancy in the study area. Social factors like caregivers’ faith and religion, patriarchy, negative experiences and doubts of caregivers and their access to the internet and social media influence the trust of caregivers. Furthermore, anti-vaccine influencers affect caregivers’ decisions through extensive utilization of social media platforms like Facebook, WhatsApp and YouTube to propagate their ideas. As reported in many other contexts, health system-related factors also influence the complex issue of resistance to immunization and the trust of caregivers in these programmes. Here, the general lack of trust in public healthcare services, which is dominated by allopathic medicine, is extended to the realm of immunization services. The inability of the healthcare workers to clarify queries about vaccination adds to a furthering of the mistrust of caregivers.

Studies conducted around the world, including India, have demonstrated that religion and faith are key players in the decision to vaccinate [26, 27]. However, our study results show that the individual’s faith and religion were not definitive factors determining decision-making on vaccination. It was apparent that vaccine resistance-related beliefs exist in the community, with one of the most prominent being the belief that vaccination is part of a depopulation agenda for certain communities, with differential vaccine vials for different communities. It is notable that external forces, such as any authority, advisors or technology, seemingly do not influence certain resistant areas; rather, these areas are inherently resistant and their inhabitants hold a different worldview on health and medicine compared to other parts of Kerala characterized by high vaccination coverage [28]. Past negative experiences also play a key role in the decision to vaccinate. Most parents are concerned about the side-effects of vaccination, along with its impact on the natural immunity of children. These concerns are largely a product of their interactions with indigenous practitioners and naturopaths and contribute to vaccine resistance and hesitancy, as reported in previous studies [29, 30].

Milieu of vaccine-related doubts prevail in the global media [31, 32]. It is also reflected in the local media, which has increased vaccine resistance among caregivers. Another key factor is the influence of patriarchy in the decision to vaccinate [33]. This may also be linked to religion, although only in a limited sense because it is largely a social reality that fathers are the decision-makers in many families and therefore patriarchal norms cut across religions. In most cases, these are absentee fathers who work in Middle Eastern countries who obtain their information against vaccination through the internet and social media, such as Facebook and WhatsApp [34]. Since they are casual visitors to the district, health workers do not have the opportunity to communicate with them and often the well-educated and well-off mothers are not able to convince the absent fathers and the present grandfathers of the household to give permission for child vaccination.

In this context, the influx of anti-vaccine messages that are widely accessible through social media is a key factor [35, 36]. Anti-vaccine groups, including naturopaths and homeopaths, use retracted and withdrawn journal articles from the past and edited and manipulated videos that highlight trivial and known short-term adverse effects, such as fever, excessive crying and restlessness, as well as non-associated problems, such as paralysis, allergy, tiredness and weakness, and use them as arguments to abstain from vaccination. Such negative messages are widely circulated by social media. At the same time, anti-vaccine activists in Kerala also exhibit “denialism”, as explained by Diethelm and McKee [36]. Anti-vaccine lobbyists use “rhetorical arguments to give the appearance of legitimate debate and unresolved debate about matters generally considered to be settled” and in the process, use and create untrue reports, views, expert opinions and results to maintain the “conspiracy theory” regarding vaccination [31, 32, 37]. In our study, an important driver that contributed to mistrust is the role of naturopathy and homeopathy, the practitioners of which openly criticize vaccination. Parents who adhere to such systems are given wrong information to create an impression that allopathic medicine has many side-effects, which in turn leads to mistrust. This has also been reported in other parts of the world [38].

From the service delivery point of view, we noted that there is a lack of trust in the public health sector, at all levels of care. Parents expressed concerns about vaccine safety, especially related to vaccination storage during mass vaccination campaigns. Lack of trust was worsened when health workers (ASHAs and Anganwadi workers) were unable to answer the questions and concerns of caregivers. In general, health workers are not trained nor prepared to manage difficult conversations with vaccine-resistant caregivers, have a limited knowledge of vaccines and miss the specific training needed to handle such situations, all of which contribute to trust issues [38]. Respondents felt that the health workers reach out only on vaccine-related matters, which creates mistrust. Doubts persist as to why the government is ‘so overtly interested’ in only vaccinating the children and not in other issues of public health relevance. Heightened campaigns by the public health system staff during mass vaccination programmes also led to suspicions and hesitancy among the caregivers. In the local setting, allopathic practitioners are largely curative oriented, giving less priority to healthy life-style practices such as exercise, walking, etc. This leads to the wrong impression that such healthy practices belong to the alternate systems of medicine. Furthermore, there is lack of coordination between different systems of medicine within the government health system, especially when practitioners of certain alternative systems have a different viewpoint on vaccines.

### Limitations of the study

The results of the study are relevant to countries and contexts with relatively higher internet coverage and social media usage. Most of the health workers we interviewed belonged to the public sector. However, in most healthcare systems, immunization is a service largely delivered by the public sector. Furthermore, we collected data from all relevant stakeholders at the household level (fathers, mothers, grandfathers and grandmothers) which should have provided us with sufficient information on the demand side factors in the decision-making process.

## Conclusions

The trust deficit between parents/caregivers and healthcare providers is created by multiple factors, such as providers’ lack of technical knowledge, existing patriarchal societal norms and the critical view of vaccines held by naturopaths and homeopaths. Anti-vaccine groups use social media to influence caregivers' perceptions and beliefs. Religion does not appear to play a major role in creating vaccine resistance in this setting.

Addressing the issue of the trust deficit is a challenging task and requires a multipronged, long-term strategy. However, we propose four practical interventions from the health sector point of view that can give results in the short to medium term. First, develop appropriate and targeted vaccine-related communication strategies, including the use of infographics, that can be shared through social media platforms. Secondly, enable healthcare workers by providing soft skill training programmes so that they can address the anti-vaccine propaganda with confidence. Thirdly, enhance the technical competence of healthcare workers on vaccines through a mobile application-based repository of information and frequently answered questionss. Finally, start a social media cell for the Department of Health with the responsibility to monitor vaccine-related conversations in social media and intervene if needed.

## Supplementary Information

**Additional file 1.** Summary of the themes, underlying factors and examples of narratives from the qualitative interviews.

## Data Availability

All data analyzed during this study are included in this published article (and Additional file 1).

## Abbreviations

ASHA: Accredited social health activist
CHC: Community health centers
DMO: District Medical Officer
FGD: Focus group discussion
HSC: Health subcentre
IDI: In-depth interview
IEC: Information education communication
NFHS: National Family Health Survey
NIP: National Immunization Programme
PHC: Primary health centres
VPD: Vaccine-preventable diseases
