# Supplementary material for: Social media, vaccine hesitancy and trust deficit in immunization programs: a qualitative enquiry in Malappuram District of Kerala, India
Source: Health Res Policy Syst. 2021 Aug 11;19(Suppl 2):56. doi: 10.1186/s12961-021-00698-x (PMC8356375; doi:10.1186/s12961-021-00698-x)
Supplement: Supplementary file 1 — Additional file 1. Summary of the themes, underlying factors and examples of narratives from the qualitative interviews. [file 12961_2021_698_MOESM1_ESM.docx]

**Additional file 1: Summary of the themes, underlying factors and examples of narratives from the qualitative interviews**

| Category | Theme | Underlying factors denoted | Example narratives |
| --- | --- | --- | --- |
| 1. Social factors | Issues related to faith and religion | Religious beliefs has limited role | *“Religion has no influence on vaccination.”*  *–*FGD, Mother of unvaccinated child, Study area 1  *“Religion doesn’t have much role in vaccination decision. People in mosque and madrassa never talk about vaccination either positively or negatively. Especially in these premises.”*  – FGD, Grandfather of unvaccinated child, Study area 1  *“Certain musaliyars (Muslim religious teachers) are against vaccination. They neither get vaccinated nor allow others in their influence to take vaccine.”*  – FGD, ASHAs, Study area 2 |
|  | Role of patriarchy and decision-making | Men are the decision makers; have, over time, been against vaccination | *“A Few women have immunized their children without the knowledge of their husband. They are terrified of someone finding out. It is normally because the child’s father or grandparents disallow them from being immunized, or they may not be educated enough.”*  – IDI, Medical officer, Study area 2  *“Some families are not taking vaccination across generation. Those families are usually following the naturopathic methods.”*  *–* IDI, Private modern medicine doctor, Study area 1  *“Many wives give vaccination to their children when husbands are [doing migrant work] at Gulf. When husbands are back home, they stop taking vaccination.”*  - FGD, ASHA, Study area 2  *“The social structure is also a reason. Highly educated girls get married to men who haven’t passed higher secondary school. Most of these men are working in Gulf countries and have good financial status. But they are against vaccination. So even though the mothers want to give vaccination the fathers won’t allow it, believing in such naturopaths (who spread the messages against vaccination through social media).”*  – IDI, District Medical Officer 1  *“One problem is mothers are the only group coming for classes. We don’t know whether this information is reaching to other people in household especially fathers.”*  – FGD, Public health nurses, Study area 2  *“ The main constraint here is[that] 60 to 70 per cent of the male population who are the decision makers are abroad. They come and go at irregular intervals. Because of that the health workers are unable to communicate with them.”*  *–*  IDI, District Medical Officer 2 |
|  | Past negative experiences and doubts | Vaccination side effects to someone in family/ distant relation; heard of adverse events following immunisation through someone or through media; vaccine ineffectiveness | *“The ill effects my elder child suffered following vaccination are the main reason for me not taking my younger children for vaccination.”*  – FGD, Father of unvaccinated child, Study area 1  *“My eldest child got Typhoid and husband got TB even after taking vaccination. That’s why I didn’t give vaccination to my youngest child. If health workers come for vaccination, I would say no to vaccination for my children. Vaccines are not safe. Diseases will come even after taking vaccination. Vaccines are not kept in proper temperature .”*  –FGD, Mother of unvaccinated child, Study area 1  *“One of my cousin’s leg was paralysed after taking polio vaccination. After that incident, no one in my family has taken vaccination.”*  –FGD, Mother of unvaccinated child, Study area 1  *“My child had fever and pain in legs after giving a vaccination. We gave that vaccination due to the doctor’s compulsion. After that I decided not to give remaining vaccination to my child.”*  *-*FGD, Father of unvaccinated child, Study area 2  *“For me, the main reason is children will get fever. And they (health workers) are not completely assuring that our child will not get affected with the disease. None in my family, including me, took vaccination…. All these things (vaccines) have become common only recently.”*  - FGD, Mother of unvaccinated child, Study area 2  *“I saw in WhatsApp that 5 children got paralyzed after taking vaccination. My friend’s one leg got paralyzed after taking polio vaccination. He had no problem till 2 years old. After taking polio vaccination his leg got paralyzed.”*  - FGD, Father of unvaccinated child, Study area 1 |
|  | Access to internet and social media | More trust in unverified sources than authentic information; Using online sources to confirm information obtained via doctors and health workers; Anti-vaccine news and programmes appearing on television | *“The vaccination dates are known through health workers. The side effects of vaccination are known through people…through WhatsApp, Facebook and newspapers…. Facebook has lot of stories about vaccine side effects. Recently in some newspaper, it was reported that three children died due to Measles Rubella vaccination… We are hearing that people are adversely affected due to vaccination and also there are news that vaccination creates health issues… Seeing all these, we are frightened. ……”*  - FGD, Mother of unvaccinated child, Study area 2  *“I am using Wikipedia and internet for searching information about medicines.”*  - FGD, Father of unvaccinated child, Study area 1  *“I usually recheck the data in internet to affirm its authenticity.”*  -FGD, Father of unvaccinated child, Study area 2  *“Now the patient is coming to test the knowledge of doctor. They will search the details of medicine (we prescribe) in internet.”*  *–* IDI, Ayurveda doctor, Study area 1  “A TV *channel telecasted a program about the hidden agenda behind vaccination. It became viral here and it has become the major source of messages.”*  -FGD, Father of unvaccinated child, Study area 2  *“One of the main reasons for resistance is TV and newspapers. They are not at all supportive to vaccination. They are very much interested in reporting a death followed by vaccination. They don’t even search the medical aspects behind it. There are also local media who propagate false information.”*  – FGD, Doctors, Study area 2 |
| 2. Anti-vaccine influencers | Local groups and individuals | Local anti-vaccine groups actively promote anti-vaccine messages through social media | *“Government has a hidden agenda to suppress news which are against vaccination (in newspapers and television)…Because of this we are forced to depend on alternative media. Social media become popular in the last 2-3 years.”*  – FGD, Father of unvaccinated child, Study area 2  *“I can say the social media has a huge power to conquer the human mind. The penetrating power of WhatsApp message is very high compared to any other social media…because each individual is the member of 2 or 3 WhatsApp groups. So, the spread of negative messages are very high among the groups or between the groups. The circulation of negative messages is high when compared to the positive message about vaccination. I haven’t seen single message advising to take vaccination.”*  – IDI, Medical Officer, Study area 1 |
|  | Social media groups | Anti-vaccine messages through social media (such as depopulation, international lobbying, harmful vaccine contents); Trust in social media messages than information provided by government sources, Impact created by video, audio and text messages | *“People have a fear of death and some says that vaccination is to depopulate us. Also children face side effects after vaccination. There are many who [think] that government has some hidden agenda behind vaccination.”*  – IDI, Muslim religious head, Study area 2  *“The main concern is that this is something imported from abroad which is not safe for children. That may be true or false. The vaccines might be scientifically proven. But in my perspective, these vaccines are coming from the USA and other foreign nations which they are forcefully trying to use in our children.”*  – FGD, Grandfather of unvaccinated child, Study area 1  *“Messages say that vaccines are not error free and they will cause some other diseases. They are also saying that vaccination is Bill Gates depopulation agenda to reduce the world population from 7 billion to 1 Billion! Vaccination is a part of international lobby….”*  -FGD, Father of unvaccinated child, Study area 2  *“Also there are arguments about the production and content of vaccination. Many saying that it has mercury content, which would cause autism in children. Though I do not believe in such things, I think such things should be more transparent regarding the production and content of vaccination. I think people are ignorant about all these things, hence they are confused.”*  –IDI, Ayurveda Doctor, Study area 2  *“The negative messages about vaccination are explaining with rationale, scientific explanations, visible examples and illustrations. They are more explanatory, rational and have proofs (live examples) also. They are projecting the consequences/side effects of vaccination.”*  –FGD, Father of unvaccinated child, study area 1  *“But negative messages get more visibility and circulation all times. People usually believe in negative messages which have no scientific background at all.”*  *–* IDI, Communication expert  *“Two audio clips are circulating in social media. It is actually a telephone call. The one who is speaking is [said to] have an autistic child and he says his child became autistic after vaccination. He is presenting all evidences too. I haven’t seen any messages which projected evidences to counter these so called negative messages.”*  – FGD, Father of unvaccinated child, Study area 1  *“Audio voice started with saying that he is a doctor and vaccines are made from dog’s kidney.”*  –FGD, Mother of unvaccinated child, Study area 1 |
| 3. Service delivery factors | Lack of trust by caregivers | Lack of trust in allopathy; Doubts about contents and quality of vaccines; Concerns about vaccine safety; Doubt in doctors and health department; Lack of communication between doctors and caregivers | *“We are consulting homeo/ Ayurveda for all health problems… We have no trust in allopathic. Allopathic medicines are not safe…”*  –FGD, Mother of unvaccinated child, Study area 1  *“I am concerned about vaccination storage. Whether it is properly refrigerated? I am also concerned about immediate side effects of vaccination like fever. Vaccine related messages are not saying not to take vaccination, but it’s telling about the risk in vaccination.”*  –FGD, Mother of unvaccinated child, Study area 1  *“Actually I have no tensions/fear about vaccination. The authority, which implements the vaccination campaign, should give an assurance in a signed stamp paper about 100% protection from any of the side effects or the incidence of vaccine preventable diseases after vaccination.”*  – FGD, Father of unvaccinated child, Study area 1  *“Doctors said that some side effect will be there for one in 1000 children? What if that one child is ours?”*  -FGD, Father of unvaccinated child, Study area 1  *“I have spoken to health workers who have compelled me to give vaccination. They came for polio vaccination. When I asked them about the mercury content in polio vaccine, they told that is in old polio (vaccine), new polio (vaccine) doesn’t have any. I asked her that how she can be that sure that the new polio vaccine doesn’t have mercury content in it. She just left saying that “you people won’t change.” She didn’t clarify the doubts.”*  - FGD, Mother of unvaccinated child, Study area 2 |
|  | Factors related to health workers | Inability of health workers to articulate properly; Lack of knowledge of health workers; Doubt in the knowledge and integrity of modern medicine doctors; Lack of coordination between different systems of medicine; Excessive interest by the health department in giving vaccinations | *“Health workers are actually servants of people… But they don’t take care of the basic health issues in a locality they are serving but they are very much interested in giving vaccination…. This is suspicious. …This makes people definitely think why they are more interested in vaccination than the more important public health issues in the locality…for a healthy life, clean food, air and water is essential. Our health department doesn’t have any role providing these three. And they are not interested in taking action against people who are polluting food, air and water. But they have great interest in giving vaccination… Because of this people do not believe the health workers…”*  - FGD, Father of unvaccinated child, Study area 2  *“They (health workers) didn’t explain the negative effects of the particular vaccine at any stage of vaccination program.”*  – FGD, Father of unvaccinated child, Study area 1  *“There is no conscious effort from the side of health department to take care of this matter and convince us about the relevance of vaccination.”*  - FGD, Father of unvaccinated child, Study area 1  *“People are interacting only with ASHA and Anganwadi workers who don’t have high level information. They have only information which they got from doctors and other high officials. Common people these days have more information than them, so the authenticity of these health workers is under question. People tend to think that health workers are saying these things as a part of their duty. Same is the case with doctors’ advice too. People have a notion that doctors are getting commission for each and every medicines they prescribe.”*  - FGD, Father of unvaccinated child, Study area 2  *“Actually we can’t convince the laymen about the science behind vaccination. It is very difficult and complex. Workforce is not trained and equipped in vaccination program. We need more classes about vaccination, how it works in the body and the manufacturing company of vaccine in simple words.”*  – FGD, ASHA, Study area 1  *“Quality of ASHA worker should be improved through training and updating the information.”*  – FGD, Fathers of vaccinated children, Study area 1  *“People are not enquiring whether these doctors are original or quack. When JPHNs and JHI go for field visit, people counter them using this particular point that “doctors are saying not to take vaccination, you people are just nurses, why do we believe you”. For example the case of Mr.X, he is known as a naturopathy doctor, but he doesn’t have any medical degree. People came to know the real colour of this man during the recent MR vaccination because of various cases registered against him. They blindly believed and still believing him though.”*  – IDI, District Immunization Officer  *“People have the tendency to think everything ‘natural’ is good. Naturopaths are flourishing like Godmen. They believe that natural things will never harm their health. So this mentality makes them to follow the advices given [by] Naturopaths blindly.”*  *–* IDI, Private modern medicine doctor, Study area 1  *“Naturopathy doctors describes various diseases attributable to vaccines”*  – IDI, Muslim religious head, Study area 1 |
